# T-cell libraries allow simple parallel generation of multiple peptide-specific human T-cell clones

**DOI:** 10.1016/j.jim.2016.01.014

**Published:** 2016-03

**Authors:** Sarah M. Theaker, Cristina Rius, Alexander Greenshields-Watson, Angharad Lloyd, Andrew Trimby, Anna Fuller, John J. Miles, David K. Cole, Mark Peakman, Andrew K. Sewell, Garry Dolton

**Affiliations:** a Division of Infection and Immunity, Cardiff University School of Medicine, Cardiff, UK; b QIMR Berghofer Medical Research Institute, Brisbane, Queensland 4029, Australia; c Peter Gorer Department of Immunobiology, King's College London Faculty of Life Sciences & Medicine, Guy's Hospital, London, UK

**Keywords:** APC, Antigen presenting cells, CDH3, cadherin-3, ^51^Cr, chromium-51, DC, dendritic cell, EBOV-Z, Zaire Ebola virus, EBV, Epstein–Barr virus, ELISA, enzyme-linked immunosorbent assay, ELISpot, enzyme-linked immunospot assay, EN2, Engrailed-2, flu, influenza A, FBS, foetal bovine serum, GAD65, glutamic acid decarboxylase, gp, glycoprotein, HA, haemagglutinin, HLA, human leukocyte antigen, IGRP, islet-specific glucose-6-phosphatase catalytic subunit-related protein, IMP-3, Insulin-like growth factor 2 mRNA binding protein 3, InsB, insulin β chain, MAGE, melanoma-associated antigen, MHC, major histocompatibility complex, MP, matrix protein, NP, nucleoprotein, PAP-3, prostatic acid phosphatase-3, PBMC, peripheral blood mononuclear cells, PHA, phytohemagglutinin, pMHC, peptide–MHC, PPI, preproinsulin, SFC, spot forming cells, TCR, T-cell receptor, T1D, Type 1 diabetes, Ebola, Library, Peptide-specific, T-cell clone, Tumour, Type 1 diabetes

## Abstract

Isolation of peptide-specific T-cell clones is highly desirable for determining the role of T-cells in human disease, as well as for the development of therapies and diagnostics. However, generation of monoclonal T-cells with the required specificity is challenging and time-consuming. Here we describe a library-based strategy for the simple parallel detection and isolation of multiple peptide-specific human T-cell clones from CD8^+^ or CD4^+^ polyclonal T-cell populations. T-cells were first amplified by CD3/CD28 microbeads in a 96U-well library format, prior to screening for desired peptide recognition. T-cells from peptide-reactive wells were then subjected to cytokine-mediated enrichment followed by single-cell cloning, with the entire process from sample to validated clone taking as little as 6 weeks. Overall, T-cell libraries represent an efficient and relatively rapid tool for the generation of peptide-specific T-cell clones, with applications shown here in infectious disease (Epstein–Barr virus, influenza A, and Ebola virus), autoimmunity (type 1 diabetes) and cancer.

## Highlights

• Methodology allows isolation and cloning of multiple T-cell clones in parallel
• Simple and requires ~ 6 weeks to validated clones
• No requirement for peptide–MHC multimers or single cell sorting
• CD4 + and CD8 + T-cell clones grown and validated
• Clones produced for viral, autoimmune and cancer epitopes

## 1 Introduction

Classical CD8^+^ (cytotoxic) and CD4^+^ (helper) T-cell subsets scan for anomalies in the proteome by recognising peptides presented by major histocompatibility complex class I (MHC-I) and class II (MHC-II) proteins, respectively, through their heterodimeric αβ T-cell receptors (TCRs) (Attaf et al., 2015b). The TCR repertoire of a person is clonotypically diverse (Attaf et al., 2015a), with individual clonotypes exhibiting peptide cross-reactivity (Wooldridge et al., 2012, Sewell, 2012), thereby enabling the host to combat invading pathogens and occasionally neoplasms. T-cells can also play a detrimental role in host health, as is seen during autoimmune disease (Gomez-Tourino et al., 2015, Salou et al., 2015) and organ transplant rejection (Lin et al., 2015).

T-cell clones provide a good experimental system to address research hypotheses without the ambiguities associated with polyclonal populations of T-cells, and also enable production of monoclonal T-cell receptors (TCRs) for immunotherapy approaches. However, generation of T-cell clones can be challenging, with factors such as sample availability, clonotype frequency, and access to suitable detection systems all impinging on the isolation of desired peptide-specific T-cell populations.

Here we describe a library-based strategy for the relatively rapid generation of peptide-specific human T-cell clones from polyclonal populations of CD8^+^ or CD4^+^ T-cells. Firstly, CD3/CD28 microbeads were used to amplify the T-cells (Trickett and Kwan, 2003) in a 96U-well library format, from which they were subsequently screened for reactivity against desired peptides via enzyme-linked immunospot assay (ELISpot). T-cells from peptide-reactive wells were then enriched using cytokine-mediated strategies, subjected to single-cell cloning, and grown to sufficient numbers for functional validation, with the entire process from blood to validated clone taking as little as 6 weeks. We have now used this T-cell library approach to generate many hundreds of different T-cell clones, without the need for access to peptide-MHC (pMHC) multimers or autologous dendritic cells (DCs). Our methodology is not only compatible with small sample sizes (e.g. 1 ml of blood or a small biopsy), but also permits the isolation of potentially rare T-cell clonotypes from diverse polyclonal T-cell populations. Overall, T-cell libraries represent a simple and efficient tool for the simultaneous detection and isolation of multiple peptide-specific T-cell clones, with examples shown here for infectious disease (Epstein–Barr virus, influenza A, and Ebola virus), autoimmunity (type 1 diabetes) and cancer.

## 2 Materials and methods

### 2.1 T-cell subset isolation

Buffy coats from healthy human leukocyte antigen (HLA)-A*0201^+^ (HLA-A2^+^) donors were obtained from the Welsh Blood Service. Peripheral blood was obtained from a healthy HLA-DRB*0101^+^ (HLA-DR1^+^) donor, an HLA-A2^+^ donor with type 1 diabetes (T1D), and a healthy HLA-A2^+^ donor who had previously participated in a clinical trial for an Ebola virus (EBOV) DNA vaccine (EBODNA012-00-VP) (Martin et al., 2006). Informed consent was obtained from all donors, and blood was collected according to institutional guidelines. Isolation of peripheral blood mononuclear cells (PBMC) was carried out by density gradient centrifugation. On day 1 of the described method, T-cells were enriched from fresh or frozen PBMC by positive selection with anti-CD8^+^ or -CD4^+^ microbeads, according to the manufacturer's instructions (Miltenyi Biotec, Bergisch Gladbach, Germany).

### 2.2 Tumour lines and immortalised cell lines

All HLA-A2^+^ tumour lines (Mel 624, Mel 526, MM909.24 and MCF-7) were maintained in culture at 37 °C as adherent monolayers with R10 media (RPMI 1640 medium supplemented with 10% foetal bovine serum (FBS), 100 U/ml penicillin, 100 μg/ml streptomycin, and 2 mM l-Glutamine (Life Technologies, Paisley, UK)). T2 cells expressing either HLA-A2 (T2) or T2 cells transduced with HLA-DR1 (T2-DR1s) were cultured at 37 °C as suspension cells in R10.

### 2.3 Production of HLA-DR1 expressing T2 cells

HLA-DR1 was cloned into the pRRLSIN.cPPT.PGK-GFP.WPRE transfer vector (Addgene #12252). Integrase proficient lentivirus stocks were produced by co-transfecting 293T/17 cells via calcium phosphate precipitation with the transfer vector and packaging plasmids: pCMV-dR8.74 (Addgene #22036) and pMD2.G (Addgene #12259). Lentivirus containing supernatant was collected after 24 h and 48 h incubations. The lentivirus stocks were concentrated by ultracentrifugation before being used to transduce T2 (174 × CEM) cells. The surface expression of DR1 was assessed using a mouse anti-human unconjugated HLA-DR antibody (clone L243, 0.5 mg/ml; Biolegend®, London, UK) and a goat anti-mouse polyclonal conjugated antibody (BD Biosciences, Oxford, UK). Populations were then enriched using the above antibodies and anti-fluorochrome microbeads (Miltenyi Biotec). Monoclonal populations were generated by single-cell cloning of the HLA-DR1 enriched population.

### 2.4 Establishing CD8^+^ and CD4^+^ T-cell libraries

**Fig. 1:**
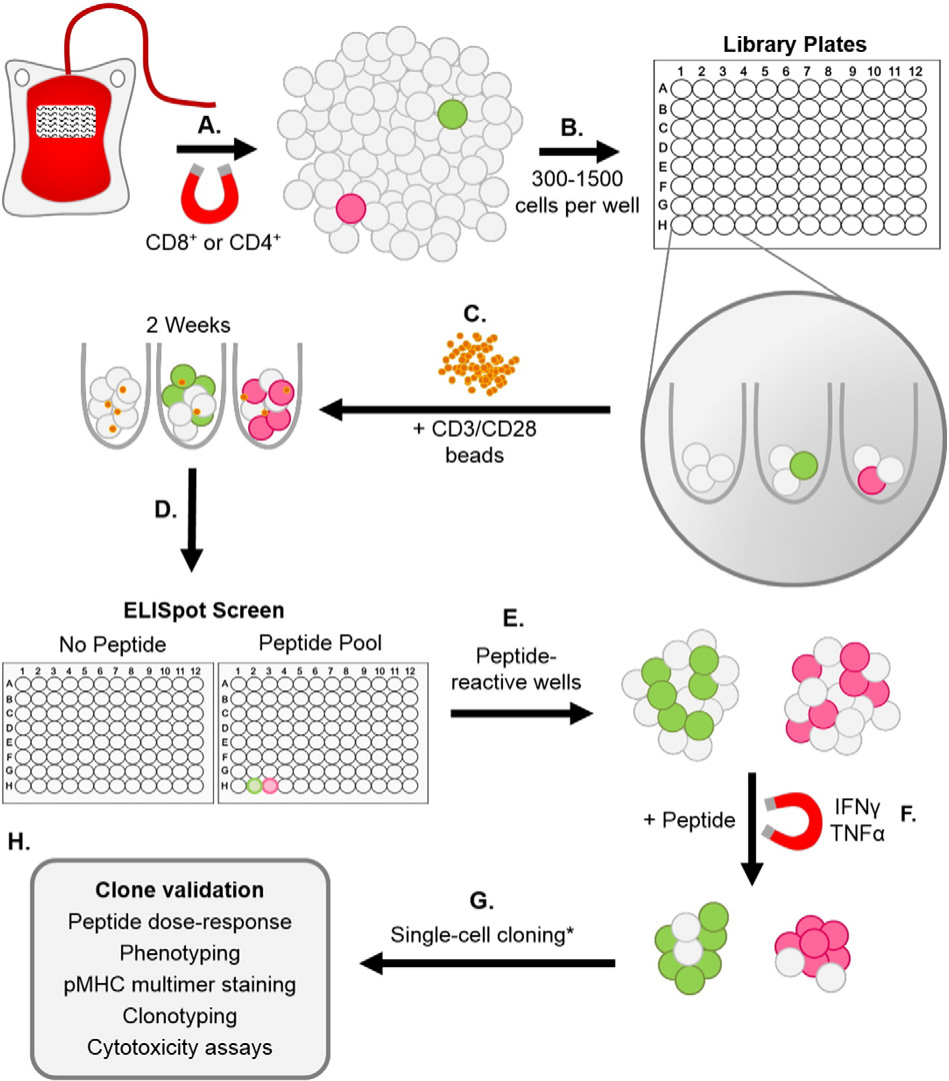
Overview of T-cell library methodology. (A–C) T-cells were enriched from fresh or frozen peripheral blood mononuclear cells (PBMC) via magnetic separation using anti-CD8 ^+^ or -CD4 ^+^ microbeads, prior to seeding into multiple 96U-well plates (range tested from 300–1500 cells per well) with CD3/CD28 beads at a 1:2 cell:bead ratio. (D) Approximately 2 weeks after initial T-cell activation with the beads, libraries were screened ± peptide(s) by IFNγ enzyme-linked immunospot assay (ELISpot). (E & F) Peptide-reactive wells identified from the screen were then enriched for peptide-specific T-cells using either an IFNγ or dual IFNγ/TNFα capture method. (G) T-cells were then cloned to the single-cell level * or expanded as a line. (H) Clone validation was performed by peptide titration (dose–response), phenotyping, pMHC multimer staining, clonotyping and cytotoxicity assays.

An overview of our T-cell library methodology is illustrated in Fig. 1. Following enrichment from PBMC on day 1, T-cells were immediately seeded (range of densities tested from 300 to 1500 cells per well) across multiple (typically 1 to 6) 96U-well plates with Human T-Activator CD3/CD28 Dynabeads® (Life Technologies) (Trickett and Kwan, 2003) at a 1:2 cell:bead ratio, in 20 IU IL-2 T-cell media (R10 media supplemented with 1X MEM non-essential amino acids, 1 mM sodium pyruvate, 10 mM HEPES buffer (Life Technologies), and 20 IU/ml IL-2 (aldesleukin, brand name Proleukin®; Prometheus, San Diego, CA). Library plates were spun before culture at 300 G for 5 min, and then maintained (at 37 °C) by feeding on days 3 and 6 with 20 IU and 200 IU IL-2 T-cell media, respectively. From day 9 onwards, libraries were maintained by feeding every 3 days with 200 IU IL-2 T-cell media (+ 25 ng/ml IL-15 (PeproTech, Rocky Hill, NJ) for CD8^+^ libraries). Between days 14 and 17 of culture, 3 random wells from each library plate were counted to establish an average T-cell number per well. Using this representative count, enough cells were removed from each library well to provide approximately 2.5 × 10^4^ cells per well for an ELISpot screen. These cells were rested in 96U well plates by washing in R0 (recipe as for R10 but with no serum), and then culturing for 24 h in R5 (recipe as for R10 but with 5% FBS). Rested library cells were then screened ± peptide(s) (10^− 5^ to 10^− 6^ M) via ELISpot, using 5 × 10^4^ antigen presenting cells (APC) (T2 or T2-DR1s) per well. This relatively high level of peptide was used in order to ensure capture of all responses, although the use of 10^− 7^ and 10^− 8^ M peptide also worked well (data not shown). We were concerned that the use of high concentrations of peptide for screening might result in the generation of T-cell clones that were only capable of recognising targets displaying high densities of cognate peptide. These worries were unfounded as the clones generated by this method were often capable of recognising lower levels of peptide, as demonstrated by the peptide titration data in the relevant figures. ELISpot screens were carried out according to the manufacturer's instructions (Mabtech, Nacka, Sweden), and an AID ELISpot reader (AID, Strassberg, Germany) was used to read the number of spot forming cells (SFC) present in each well. If the limit of detection was exceeded, and individual spots could not be accurately discerned by the reader, peptide-reactive wells were enumerated by eye. Cells from peptide-reactive wells of the screen, with a SFC increase of ≥ 20 from the corresponding “no peptide” well, were either pooled or kept as individual wells.

### 2.5 Isolating T-cell clones

Peptide-specific (IFNγ or IFNγ/TNFα secreting) T-cells were isolated from the positive library well(s) by resting the cells in R5 media (as above), stimulating the rested cells with 10^− 5^ M peptide for 4 h, and then using an IFNγ or dual IFNγ/TNFα capture method to isolate the activated T-cells, according to the manufacturer's instructions (Miltenyi Biotec). The cells were then cloned to the single-cell level by dilution, or expanded as an enriched line. T-cell clones and lines were maintained at 37 °C in either 20 IU or 200 IU IL-2 T-cell media (+ IL-15 for CD8^+^ cells). T-cells were stimulated fortnightly with 1 μg/ml phytohemagglutinin (PHA) (Alere, Cheshire, UK), in the presence of irradiated (3100 Gy) allogeneic feeder cells (PBMC) from three healthy donors (5 × 10^4^ per well).

### 2.6 Peptides

The peptides and their known HLA restriction are listed in Table 1. In addition to generating T-cell clones specific for established T-cell epitopes, we were also interested in using the technique to verify new epitopes. In this respect, we used the T-cell library strategy to test new peptide epitopes from Engrailed-2 (EN2) (Morgan et al., 2011), influenza A (flu) haemagglutinin (HA) (Babon et al., 2012), and the 5T4 oncofetal protein (Starzynska et al., 1994).

### 2.7 Clone validation

Peptide-specificity of the T-cell clones was determined by quantifying either MIP-1β or IFNγ release from peptide-stimulated T-cells in an enzyme-linked immunosorbent assay (ELISA), according to the manufacturer's instructions (R&D Systems, Minneapolis, MN). Typically, 6 × 10^4^ APC per well, 3 × 10^4^ rested T-cells per well, and 10^− 5^ M of peptide was used for each ELISA. Clone sensitivity (dose–response) was determined by titrating the peptide (ranging from 10^− 5^ M to 10^− 10^ M) in a MIP-1β ELISA. MIP-1β and IFNγ concentrations were calculated by subtracting the appropriate “no peptide” control wells. Where possible, staining with pMHC multimer (Dolton et al., 2015, Tungatt et al., 2015) was used to confirm TCR binding to peptide via HLA-A2 presentation. In the case of tumour-specific clones, a chromium (^51^Cr)-release cytotoxicity assay (Tungatt et al., 2015) (PerkinElmer, Waltham, MA) was carried out to determine if the T-cell clones were capable of tumour cell killing. Percentage (%) specific lysis was calculated using the following equation: (experimental release − spontaneous release) / (maximal release − spontaneous release) × 100.

## 3 Results

### 3.1 Generation of a T1D-relevant CD8^+^ T-cell clone from limited starting material

**Fig. 2:**
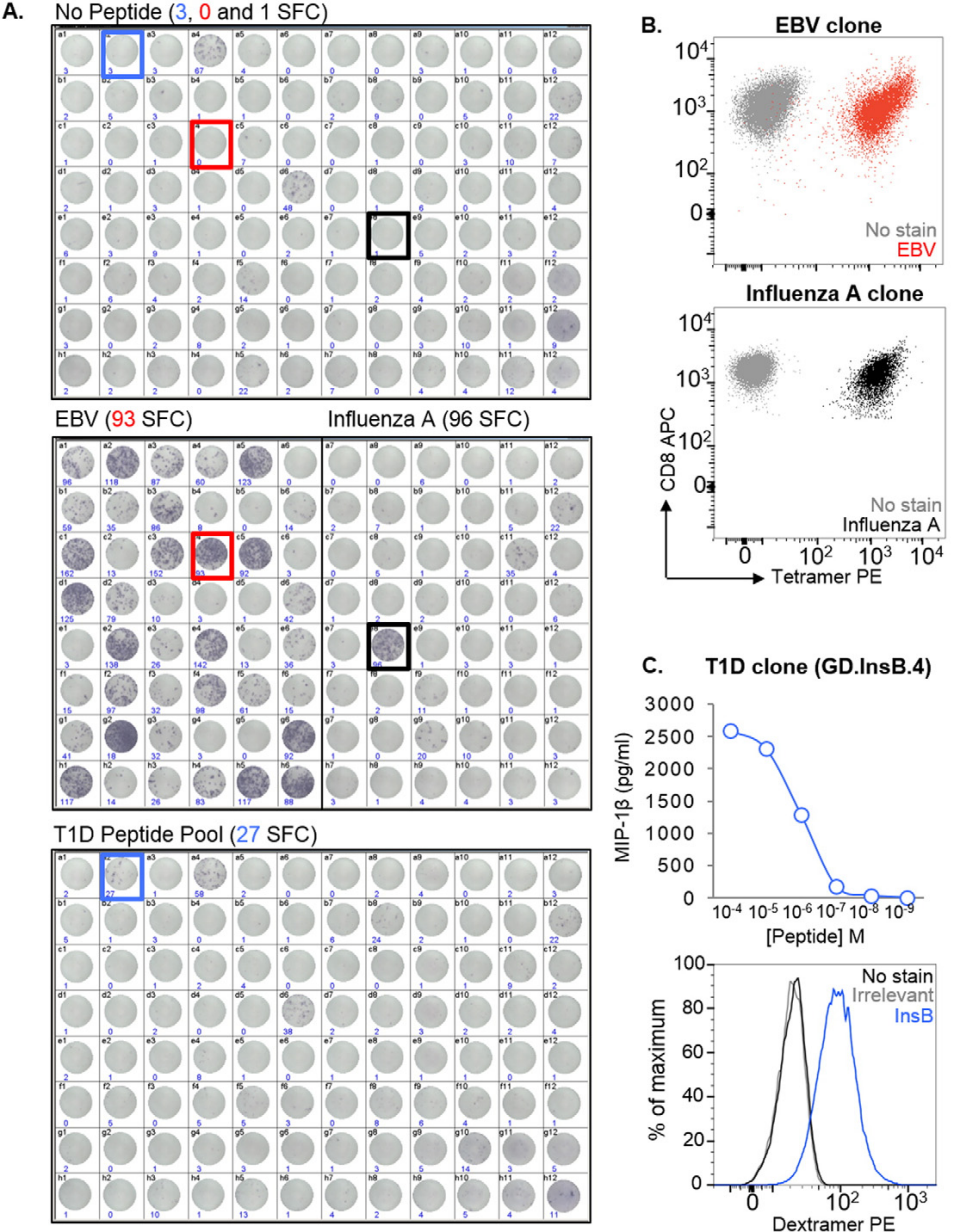
Generation of clones that recognise EBV, influenza A, and insulin β chain peptides from a type 1 diabetes (T1D) CD8^+^ T-cell library. (**A**) A CD8^+^ T-cell library (96 wells at 1000 cells per well) from an HLA-A2^+^ donor with T1D was screened by IFNγ enzyme-linked immunospot assay (ELISpot), using T2 as antigen presenting cells. Library cells were tested alone (top), against HLA-A2-restricted peptides from Epstein Barr virus (EBV) (BMFL1_280–288_) and influenza A (flu) virus (matrix protein (MP)_58–66_) (middle), and against a pool of four HLA-A2-restricted T1D-relevant peptides (preproinsulin (PPI)_15–24_, insulin β chain (InsB)_10–18_, glutamic acid decarboxylase (GAD65)_114–123_ and islet-specific glucose-6-phosphatase catalytic subunit-related protein (IGRP)_265–273_) (bottom). The number of spot forming cells (SFC) per 3.3 × 10^4^ cells, as determined by the ELISpot reader, is shown for each well. Those used for single-cell cloning following IFNγ enrichment are colour coded (EBV: red, flu: black, T1D: blue). (B) T-cells cloned from the EBV (**A**, red) and flu (**A**, black) peptide-reactive wells were stained with cognate pMHC tetramer. (**C**) A T-cell clone (GD.InsB.4) from the positive well for the T1D peptide pool (**A**, blue) was tested against individual peptides (data not shown), titrated against the InsB peptide in a MIP-1β ELISA (top), and stained with pMHC dextramer assembled with the same epitope (bottom).

One of the main challenges in identifying and isolating peptide-specific T-cells from patients is that there is often limited cell availability due to sample sharing between researchers, ethical limitations on the size/volume of the sample that can be taken, and also the nature of the tissue source, such as with biopsies. To demonstrate that T-cell libraries can be used to overcome this limitation, 1 ml of blood from an HLA-A2^+^ donor with T1D was used to make a library consisting of 96 wells with 1000 CD8^+^ T-cells per well. After 14 days, and an approximate 300- to 400-fold expansion of T-cells, the library was screened against two pools of HLA-A2-restricted peptides using IFNγ ELISpot. Peptides from Epstein–Barr virus (EBV) (BMFL1_280–288_ (Steven et al., 1997)) and flu (matrix protein (MP)_58–66_ (Bednarek et al., 1991)) were used to test the feasibility of screening in this manner, as robust T-cell responses are elicited in HLA-A2^+^ people exposed to these viruses. Background release of IFNγ without added peptide was observed in some wells (Fig. 2A, top), however this did not preclude the identification of peptide-reactive wells: 30 wells of the 48 screened (17/48) were positive for the EBV peptide, and 2/48 for the flu peptide (Fig. 2A, middle). More interestingly, 1/96 of the wells screened with a pool of four well-characterised T1D-relevant peptides (preproinsulin (PPI)_15–24_ (Skowera et al., 2008), insulin β chain (InsB)_10–18_ (Pinkse et al., 2005), glutamic acid decarboxylase (GAD65)_114–123_ (Panina-Bordignon et al., 1995), and islet-specific glucose-6-phosphatase catalytic subunit-related protein (IGRP)_265–273_ (Jarchum et al., 2008)) was peptide-reactive (Fig. 2A, bottom). One peptide-reactive well from each of the three screens was enriched based on IFNγ production in response to stimulation with relevant peptide(s), prior to T-cell cloning. pMHC tetramer staining was used to confirm the specificities of the EBV and flu clones (Fig. 2B). We established that the T1D peptide-reactive clone (GD.InsB.4) was specific for the InsB peptide, as shown via peptide dose–response (MIP-1β ELISA) and pMHC dextramer staining (Fig. 2C). All the InsB clones that were grown stained with the same TCR variable β chain antibody (data not shown), suggesting they were likely to be derived from the same precursor. Together, these data verify that peptide-specific T-cells of interest can be successfully isolated from patient samples even when cells are in short supply.

### 3.2 Generation of tumour-specific T-cell clones from potentially rare populations using CD8^+^ T-cell libraries

**Fig. 3:**
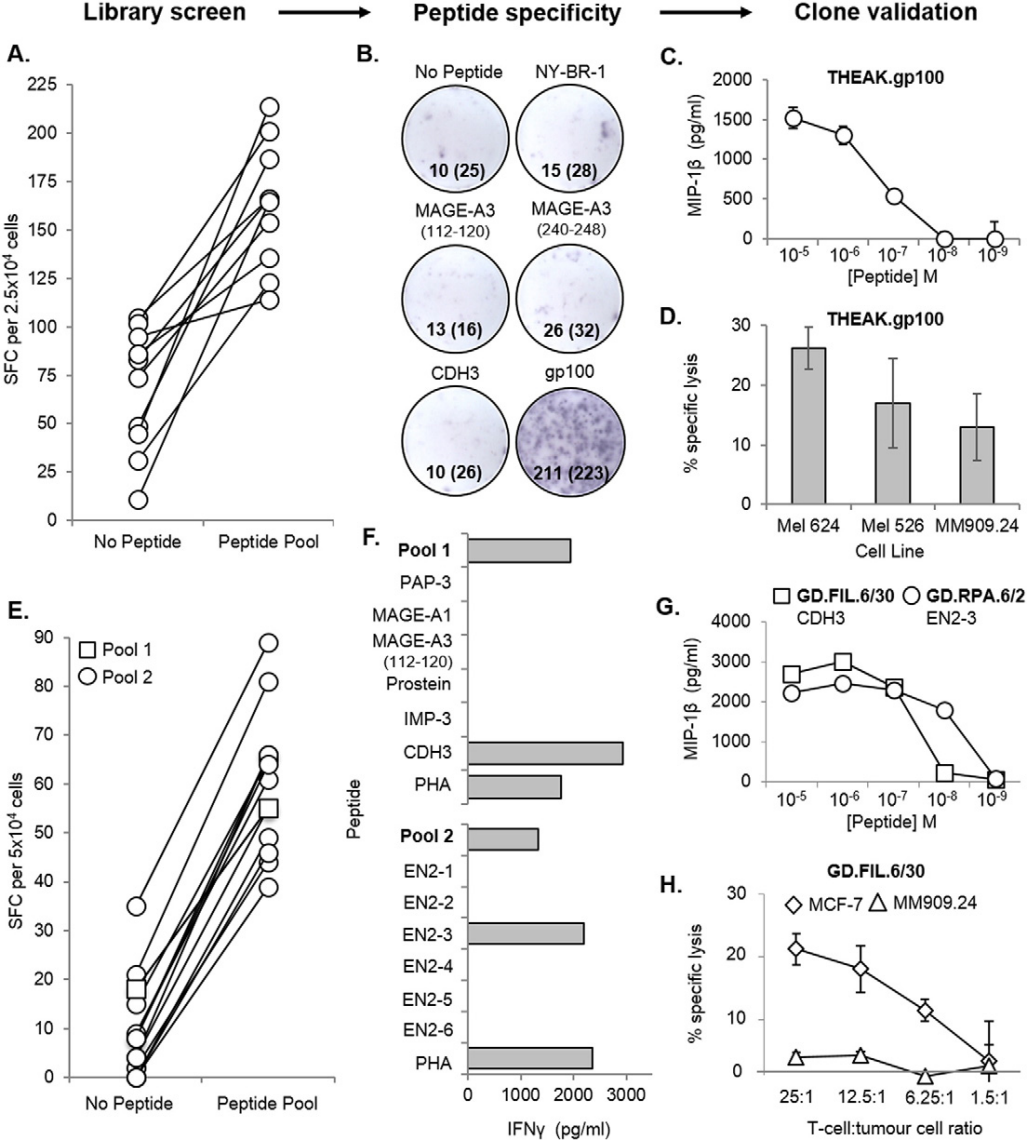
CD8^+^ T-cell clone generation from T-cell libraries screened with tumour peptides. A CD8^+^ T-cell library (576 wells at 1000 cells per well) from an HLA-A2^+^ donor was screened ± an HLA-A2-restricted tumour peptide pool by IFNγ enzyme-linked immunospot assay (ELISpot), using T2 as antigen presenting cells. (**A**) Spot forming cells (SFC) per 2.5 × 10^4^ cells is shown for the 10 peptide-reactive wells. (B) Positive wells were then pooled, and tested for individual peptide-specificity by IFNγ ELISpot. SFC per 2.5 × 10^4^ cells for each well is shown (SFC for duplicate wells has been shown in brackets). The pooled cells were then enriched for reactive T-cells based on IFNγ production, and subjected to single-cell cloning. (C & D) One of the clones (THEAK.gp100) was specific for the gp100-derived peptide by MIP-1β ELISA, and also successfully killed multiple HLA-A2^+^ melanoma cell lines (Mel 624, Mel 526, and MM909.24) in a ^51^Cr-release assay after 18 h, at a T-cell:tumour cell ratio of 10:1. A library from a second HLA-A2^+^ donor (288 wells at 500 cells per well) was screened as in (A), but with two pools of HLA-A2-restricted tumour peptides. (E) SFC per 5 × 10^4^ cells for the 14 peptide-reactive wells. (F) Cloned T-cells were screened against individual peptides by IFNγ ELISA, and were found to recognise a peptide from either cadherin-3 (CDH3) or Engrailed-2 (EN2). (G) Both the CDH3-specific clone (GD.FIL.6/30) and EN2-3-specific clone (GD.RPA.6/2) were tested for sensitivity to cognate peptide by MIP-1β ELISA. (H) The GD.FIL.6/30 clone was also tested for cytotoxicity towards an HLA-A2^+^ breast cancer cell line (MCF-7), and an HLA-A2^+^ metastatic melanoma cell line (MM909.24) in a ^51^Cr-release assay after 4 h.

In addition to the limitations associated with sample size, the production of T-cell clones is often made more difficult when the peptide-specific T-cells of interest occur at naturally low frequencies. This is often the case with tumour-reactive T-cells (Sharpe and Mount, 2015) (recognising tumour-associated antigens) in PBMC, as a result of thymic selection reducing the presence of “self” reactive T-cells in the periphery (Klein et al., 2014). Thus, with the aim of isolating rare tumour-specific T-cells, a CD8^+^ T cell library (576 wells at 1000 cells per well) was generated from the PBMC of a healthy HLA-A2^+^ donor, and screened via IFNγ ELISpot against a pool of five HLA-A2-restricted tumour peptides (melanoma-associated antigen-3 (MAGE-A3)_112–120_ (Chinnasamy et al., 2011), MAGE-A3_240–248_ (Graff-Dubois et al., 2002), cadherin-3/P-cadherin (CDH3)_655–663_ (Imai et al., 2008), NY-BR-1_904–912_ (Wang et al., 2006) and glycoprotein 100 (gp100)_280–288_ (Kawakami et al., 1995)). Fig. 3A shows the positive library wells (10/576) from this library screen, which were subsequently pooled, and specificity for the gp100 peptide determined (by IFNγ ELISpot) prior to enrichment and cloning (Fig. 3B). From this library, a gp100-specific clone (THEAK.gp100) was produced, and its reactivity confirmed via a peptide dose–response experiment using MIP-1β ELISA (Fig. 3C). THEAK.gp100 was able to kill multiple HLA-A2^+^ melanoma cell lines (Mel 624, Mel 526 and MM909.24) in a ^51^Cr-release assay after 18 h, at a T-cell:tumour cell ratio of 10:1 (Fig. 3D).

Next, a second CD8^+^ T-cell library (288 wells at 500 cells per well) was produced from a different healthy HLA-A2^+^ donor, but this time screened against two separate pools of HLA-A2-restricted tumour peptides, (Pool 1: prostatic acid phosphatase-3 (PAP-3)_299–307_ (Harada et al., 2003), melanoma-associated antigen-1 (MAGE-A1)_278–286_ (Pascolo et al., 2001), MAGE-A3_112–120_, prostein_31–39_ (Kiessling et al., 2004), insulin-like growth factor 2 mRNA binding protein 3 (IMP-3_199–207_) (Tomita et al., 2011), and CDH3_655–663_; Pool 2: six putative peptides from EN2 (EN2-1, -2, -3, -4, -5, and -6)). ELISpot data for the peptide-reactive wells (1/288 for pool 1, and 13/288 for pool 2) is shown in Fig. 3E. T-cells were then cloned from the positive wells, and screened against individual peptides by IFNγ ELISA (Fig. 3F). Two clones were produced; one recognising the CDH3 peptide (GD.FIL.6/30), and the other recognising a putative EN2-3 peptide (GD.RPA.2/30), as confirmed by a peptide dose–response (MIP-1β ELISA) (Fig. 3G). The CDH3-specific clone was shown to specifically kill an HLA-A2^+^ breast cancer cell line (MCF-7), and not an HLA-A2^+^ metastatic melanoma cell line (MM909.24; obtained from the Center for Cancer Immune Therapy, Herlev Hospital, Copenhagen, Denmark) in a ^51^Cr-release assay after 4 h (Fig. 3H). Collectively, these data show that obtaining tumour-reactive T-cell clones using this method is not hindered by predicted low clonotype frequencies.

### 3.3 Generation of T-cell clones from CD4^+^ T-cell libraries

**Fig. 4:**
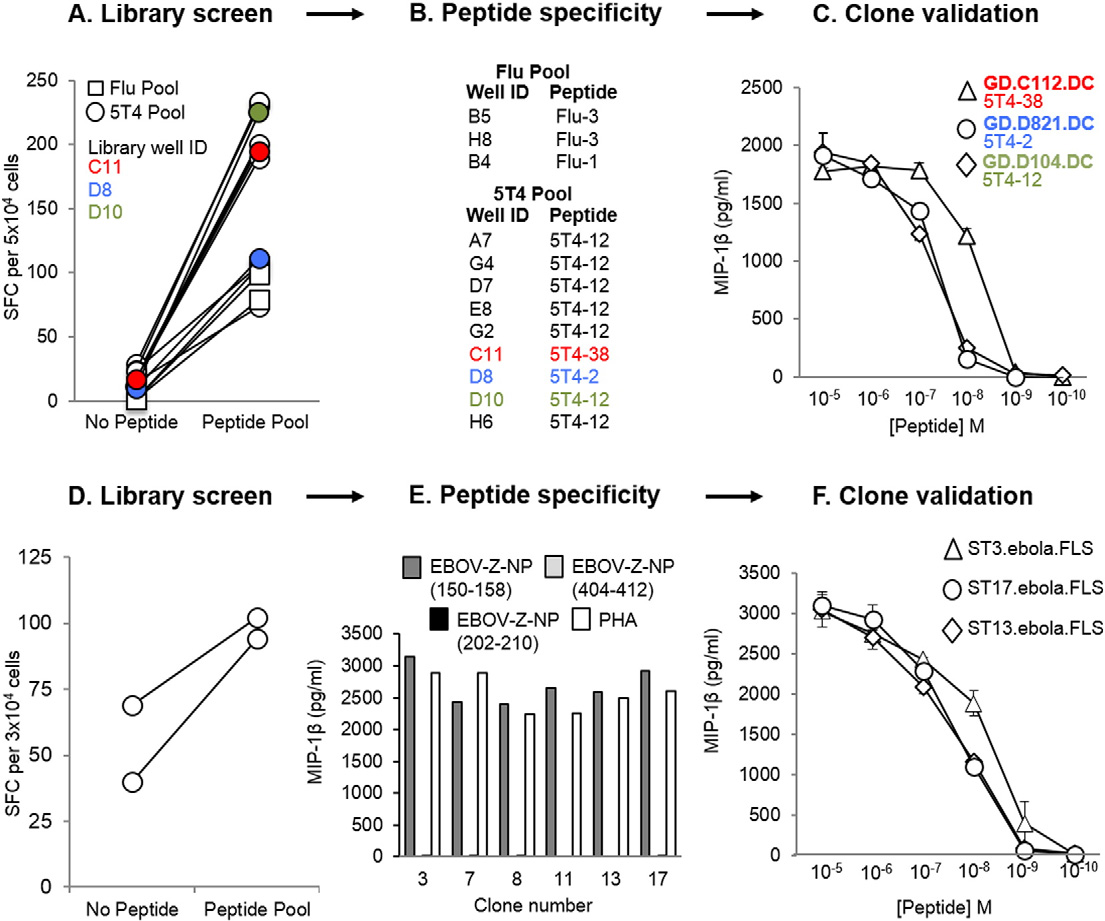
Isolation of peptide-specific CD4^+^ T-cells, and Zaire Ebola virus (EBOV-Z) specific CD8^+^ T-cells from T-cell libraries. T-cell libraries (192 wells per library at 1000 cells per well) were established from a healthy HLA-DR1^+^ donor (A–C), and a healthy HLA-A2^+^ donor who had previously participated in an EBOV DNA vaccine trial (D–F). (A) The healthy HLA-DR1^+^ library was screened by IFNγ enzyme-linked immunospot assay (ELISpot) against two pools of peptides, using T2-DR1s as antigen presenting cells (APC). Spot forming cells (SFC) per 5 × 10^4^ cells are shown for the peptide-reactive wells. 3 wells were positive for the influenza A (flu) pool (three putative peptides from haemagglutinin: Flu-1, -2 and -3), and 9 wells were positive for the 5T4 oncofetal protein pool (five putative peptides: 5T4-2, -12, -20, -38 and -PMS). 3 of the positive library wells (C11, D8 and D10), shown to respond to different 5T4 peptides, have been colour coded to illustrate their progression to validated 5T4-specific clones (B). Peptide dose–responses for the T-cell clones grown from these wells (GD.C112.DC, GD.D821.DC and GD.D104.DC) have been illustrated in (C). (D) A second library established from a healthy HLA-A2^+^ EBOV vaccinated individual was screened by IFNγ ELISpot, using T2 cells as APC. SFC per 3 × 10^4^ cells has been shown for the peptide-reactive wells. 2 wells showed a positive response to the pool of three HLA-A2-restricted epitopes (EBOV-Z-NP_150–158_, EBOV-Z-NP_202–210_, and EBOV-Z-NP_404–412_) from EBOV-Z nucleoprotein (NP). These wells were pooled, subjected to IFNγ/TNFα dual enrichment, and then cloned to the single-cell level. (E) All six clones generated a response to EBOV-Z-NP_150–158_ peptide. Dose–response curves (MIP-1β ELISA) for three of these clones (ST3.ebola.FLS, ST13.ebola.FLS and ST17.ebola.FLS) are depicted in (F).

To further illustrate the versatility of the T-cell library method, a CD4^+^ T-cell library (192 wells at 1000 cells per well), was generated from a healthy HLA-DR1^+^ donor, and simultaneously screened via IFNγ ELISpot for reactivity against two HLA-DR1-restricted peptide pools. The first peptide pool contained three putative peptides from HA of flu (Flu-1, -2, and -3), and the second peptide pool contained five putative peptides from 5T4 oncofetal protein (5T4-2, -12, -20, -38, and -PMS). Positive wells from the screen (3/48 for the flu pool, and 9/144 for the 5T4 pool: shown in Fig. 4A) were enriched based on IFNγ production in response to peptide(s), and then expanded once with PHA and irradiated allogeneic feeder cells to produce lines. The lines were subsequently screened against individual peptides in an IFNγ ELISpot (Fig. 4B), and then cloned to the single-cell level. From this, three 5T4-clones (GD.C112.DC, GD.D821.DC and GD.D104.DC) were generated and tested against decreasing doses of peptide via MIP-1β ELISA, in order to establish their sensitivity to the corresponding epitope (Fig. 4C). Thus, these data indicate that our T-cell library strategy can also successfully produce CD4^+^ T-cells with desired specificities. It is noteworthy that autologous EBV immortalised B-cell lines were initially used for the screening of CD4^+^ libraries, but these induced high numbers of positive wells (data not shown), presumably because of T-cells with reactivity against EBV.

### 3.4 Generation of Zaire Ebola virus (EBOV-Z)-specific T-cell clones from a vaccinated donor

Finally, we reasoned that this method of T-cell clone generation could also be used to generate peptide-specific T-cell clones from vaccinated individuals. Therefore, a CD8^+^ T cell library (192 wells at 1000 cells per well) was generated from the PBMC of a healthy HLA-A2^+^ donor, who had previously participated in a clinical trial for an EBOV DNA vaccine. The library was screened via IFNγ ELISpot for reactivity against a pool of three predicted HLA-A2-restricted EBOV-Z nucleoprotein (NP) epitopes (EBOV-Z-NP_150–158_, EBOV-Z-NP_202–210_, and EBOV-Z-NP_404–412_) (Sundar et al., 2007) (Fig. 4D). Positive wells from the screen (2/192) were pooled, subjected to IFNγ/TNFα dual enrichment, and then cloned to the single-cell level. Six EBOV-Z-specific clones were generated, all reactive to the EBOV-Z-NP_150–158_ peptide, as determined by MIP-1β ELISA (Fig. 4E). Peptide dose–response curves (MIP-1β ELISA) for three of the clones (ST3.ebola.FLS, ST13.ebola.FLS, and ST17.ebola.FLS) are shown as an example (Fig. 4F). These data demonstrate the ability of this T-cell library method to rapidly produce viral-specific T-cell clones from the blood of a vaccinated donor.

## 4 Discussion

Modern advances in cell sorting, using fluorescence or magnetic beads, have allowed the generation of T-cell clones following physical isolation with pMHC multimers, or functional detection using antibodies specific for cellular activation markers. Although these techniques have worked well in our laboratory for some antigens, we have failed to grow robust clones using these standard methodologies more often than we have succeeded. In order to circumvent this difficulty, we developed the T-cell library strategy described here. Previous studies have applied a T-cell library approach to study T-cell frequencies, but instead used PHA in combination with irradiated allogeneic feeder cells for T-cell expansion (Campion et al., 2014, Geiger et al., 2009). The CD3/CD28 beads used in our strategy have been shown to better preserve the TCR repertoire during *in vitro* expansion (Neller et al., 2012). Nevertheless, while this methodology maintains the general TRBV families and dominant antigen-specific T-cell responses faithfully, it remains possible that extremely rare clones are lost during this expansion phase.

Using the methodology we describe here, we have been able to simultaneously generate many hundreds of peptide-specific T-cell clones, with at least one being grown from each library. T-cell libraries have become the method of choice for generating monoclonal T-cells in our laboratory, as they avoid the need for pMHC multimers, ample donor material, or time-consuming DC production. Furthermore, we consider it an advantage to have the T-cells already adapted to *in vitro* culture prior to screening, and also to avoid repeated exposure to antigenic peptide, which can often lead to T-cell exhaustion (Wherry and Kurachi, 2015). Importantly, we have found T-cell clones to be extremely advantageous for improving pMHC multimer staining protocols (Dolton et al., 2015, Tungatt et al., 2015), T-cell epitope identification, defining T-cell cross-reactivity (Wooldridge et al., 2012), obtaining monoclonal TCRs (for genetic, biophysical and structural studies), and peptide vaccine development (Ekeruche-Makinde et al., 2012).

In summary, we have developed an efficient and reproducible library-based strategy for the successful detection and isolation of peptide-specific human T-cell clones from polyclonal CD8^+^ or CD4^+^ T-cell populations. By introducing a degree of clonality at the start of culture, and by coupling this with effective cytokine-mediated enrichment strategies, our methodology permits the relatively rapid generation of fully validated clones in as little as 6 weeks. Overall, T-cell libraries provide a useful tool for the T-cell immunologist, as they can be used for the simple parallel generation of multiple T-cell clones with numerous research applications in infectious disease, autoimmunity and cancer.

## Figures and Tables

**Table 1:** List of peptides used in this study. HLA restriction and full peptide sequence are listed in all but 3 cases. Sequences of the new HLA-A*0201-restricted Engrailed-2-derived epitopes, and the new HLA-DRB*0101-restricted epitopes from influenza haemagglutinin and 5T4 oncofetal protein will be published in other studies we are currently preparing.

Origin	Protein	Amino acid residues	Peptide sequence	HLA restriction	Reference
Epstein–Barr virus	BMLF1 lytic protein	280–288	GLCTLVAML	A*0201	Steven et al. (1997)
Influenza A	Matrix protein (MP)	58–66	GILGFVFTL	A*0201	Bednarek et al. (1991)
Influenza A	Haemagglutinin (HA)	Putative		DRB*0101	Babon et al. (2012)
Zaire Ebola virus	Nucleoprotein	150–158	FLSFASLFL	A*0201	Sundar et al. (2007)
Zaire Ebola virus	Nucleoprotein (NP)	202–210	RLMRTNFLI	A*0201	Sundar et al. (2007)
Zaire Ebola virus	Nucleoprotein	404–412	KLTEAITAA	A*0201	Sundar et al. (2007)
Type 1 diabetes	Glutamic acid decarboxylase (GAD65)	114–123	VMNILLQYVV	A*0201	Panina-Bordignon et al. (1995)
Type 1 diabetes	Insulin β chain (InsB)	10–18	HLVEALYLV	A*0201	Pinkse et al. (2005)
Type 1 diabetes	Islet-specific glucose-6-phosphatase catalytic subunit-related protein (IGRP)	265–273	VLFGLGFAI	A*0201	Jarchum et al. (2008)
Type 1 diabetes	Preproinsulin (PPI)	15–24	ALWGPDPAAA	A*0201	Skowera et al. (2008)
Tumour	Cadherin-3/P-Cadherin (CDH3)	655–663	FILPVLGAV	A*0201	Imai et al. (2008)
Tumour	Engrailed-2 (EN2)	Putative		A*0201	Morgan et al. (2011)
Tumour	Glycoprotein 100 (gp100)	280–288	YLEPGPVTA	A*0201	Kawakami et al. (1995)
Tumour	Insulin-like growth factor 2 mRNA binding protein 3 (IMP-3)	199–207	RLLVPTQFV	A*0201	Tomita et al. (2011)
Tumour	Melanoma-associated antigen-1 (MAGE-A1)	278–286	KVLEYVIKV	A*0201	Pascolo et al. (2001)
Tumour	Melanoma-associated antigen-3 (MAGE-A3)	112–120	KVAELVHFL	A*0201	Chinnasamy et al. (2011)
	Melanoma-associated antigen 3 (MAGE-A3)	240–248	YLEYRQVPG	A*0201	Graff-Dubois et al. (2002)
Tumour	NY-BR-1	904–912	SLSKILDTV	A*0201	Wang et al. (2006)
Tumour	Oncofetal protein, 5CT4	Putative		DRB*0101	Starzynska et al. (1994)
Tumour	Prostatic acid phosphatase-3 (PAP-3)	299–307	ALDVYNGLL	A*0201	Harada et al. (2003)
Tumour	Prostein	31–39	CLAAGITYV	A*0201	Kiessling et al. (2004)
